# Stem cells from human dental pulp and apical papilla: Morphological and synchrotron radiation analysis

**DOI:** 10.4317/jced.58819

**Published:** 2021-12-01

**Authors:** Karla-Mayra Rezende, Marcelo Bönecker, Luciana Côrrea, Carlos-Alberto Perez, Giancarlo-Espósito-de Souza Brito, Gabriela-Oliveira Berti, Andrea-Mantesso Pobocik

**Affiliations:** 1 Universidade de São Paulo - USP, School of Dentistry, Department of Pediatric Dentistry and Orthodontics, São Paulo, SP, Brazil; 2 Pathology Department, School of Dentistry, University of São Paulo, Brazil; 3 XRF Line, Brazilian Synchrotron Light Laboratory, Campinas, Brazil; 4 University of São Paulo, Institute of Physics, Department of Applied Physics, Rua do Matão Travessa R #187, 05508-090 São Paulo, SP, Brazil; 5 Department of Pathology, Dentistry School of University of Sao Paulo, Sao Paulo, Brazil

## Abstract

**Background:**

Dental Mesenchymal stem cells has prompted great for cell-based therapeutics. But no one knows for sure what the true potential of these cells, since most of the studies were done in isolation, using as source, different donors or different cell processing conditions.

**Material and Methods:**

An enriched population of cells positive for CD146, STRO-1, and CD90 was isolated of third molars teeth indicated for extraction of patient with of 16 years old. Analysis of cell kinetics, and subcellular tests were performed to assess the presence of minor and trace elements by using synchrotron radiation x-ray fluorescence microscopy.

**Results:**

In the cell kinetics assays, the enriched populations showed generally slower growth as compared to those that were non-enriched. In comparison between the pulp and papilla populations, the derived pulp grew more rapidly than that derived from the papilla. The CD90 + cells exhibited a smaller pulp area compared to other populations, but the papilla of these cells exhibited a larger area. The CD90 + cells exhibited higher amounts of P, S, Cl, K, and Ca, while the Cu and Zn exhibited more than CD146-. STRO1 - exhibited K and Cu. For both the pulp and the papilla, multipotent stem cells positive for all three markers were present.

**Conclusions:**

Although they have been obtained from the same tooth and donor, as well as were grown, the populations derived from these two tissues have different growth morphology and kinetics. The biochemical differences show different metabolic patterns, reflecting in part the growth differences.

** Key words:**Synchrotron radiation, dental stem cells, mesenchymal stem cells, chemical composition.

## Introduction

The maintenance of cell populations and tissue repair are made by stem cells that are capable of self-renewal and to generate daughter cells with the potential to multidifferentiation (1). These cells have been studied as a source for the development of tissue engineering with future clinical applications (2,3). However, the stem cells present in human tissues are morphologically heterogeneous and they have a short half-life after differentiation as they can no longer renew themselves(4-7).

As for the teeth, we can find adult stem cells in dental pulp, dental follicle, periodontal ligament and alveolar bone (8-11). However, it is not yet known which is the actual differentiation potential of these cells if one considers their different origins, since previous studies were separately performed by using different donors or even under different conditions of cell culture (12-14) 

Discrepancies in the detection of different makers of stem cells have been observed in the literature, indicating the need of further studies on the factors involved in the regulation of these cells(15-17). These properties range depending on the isolation/expansion method used for cell culture, which makes it difficult to compare the results from several research groups and delays the progress in this field (18,19).

Knowing the distribution of chemicals within the cells, and even within the organelles, can help reveal their activities and functioning in a variety of cellular processes (20). The study of the concentration of trace elements has played an important role in the understanding of different cellular processes. Instability of these ionic chemicals within the cells (i.e. abundance or deficiency) was recognised as being essential for the normal homeostasis of eukaryotic cells, whereas their accumulation would be potentially toxic as several diseases might be triggered (21).

Elementary chemical analysis of living matter show us that only 22 of the more than 100 chemicals are essential for living beings and of these, only 16 can be found in all species. These 16 chemicals are found in living beings at very different proportions than those of the physical milieu as carbon, hydrogen and oxygen comprise 99% of the cell mass. Sodium, potassium, magnesium, calcium, sulphur, phosphorus and chlorine represent 1-2% of the total mass of the cells. Of all the compounds, water is the most abundant in the cell, corresponding to 95% of the total mass and serving as a dispersion medium for the remaining molecules and helping in their interaction (22).

Therefore, the study of cells by means of radiation has been shown to be important because of its capacity of identifying cell components comprehensively and at high resolution. This occurs because the radiation beam can excite even small elements. Electromagnetic radiation absorbance has a wavelength ranging from 2 to 20 μm (23). For instance, hematopoietic stem cells have a mean diameter of 6 μm (24,25). Synchrotron-based X-ray fluorescence microscopy is non-invasive and non-destructive method, allowing a micron and submicron analysis without change the sample (26). From it, one may investigate the frequency profile and distribution of chemical elements in the cell interior in heterogeneous samples (23,27) and it can provide excellent effectiveness as well as good capacity of identifying and characterizing stem cells (28). It is important to highlight that up to date there is no study on mesenchymal stem cells of dental origin and synchrotron radiation in the literature.

The objective of this work was to analyse the morphological characteristics and the ionic profile of stem cell-enriched populations derived from apical papilla and pulp of permanent teeth, which were isolated by using the expression of three different markers immediately after isolation (p0).

## Material and Methods

This study assessed 16 third human molars, all with incomplete rhizogenesis indicating extraction (approved by the Research Ethics Committee of University of São Paulo, SP, n. 06/11). These teeth were immediately taken to the laboratory while being stored in a transport medium composed of 3 ml αMEM (minimum essential medium eagle - α modification, Sigma®), with 2% antibiotic-antimycotic solution (Gibco®) at the beginning of the cell culture, performed by the explant technique.

Tissues were fragmented into pieces smaller than 1 mm2 and placed in a petri dish and, were on a maintenance medium culture that consisted of αMEM (Minimum Essential Medium Eagle - α modification) with a 1% antibiotic-antimycotic solution, 1% β-mercaptoethanol and 10% fetal bovine serum (FBS), 100 uM ascorbic acid and 2 mM L-Glutamine (Glutamax®Gibco). After reaching 75% confluence on the plate, the cells were subcultured.

The cells were expanded until they reached a size of 1x107, when they were then isolated by use of the magnet (R MACS, Miltenyi Biotec) according to the manufacturer’s specifications. Thus, each line of the pulp and papilla were separated with the following markers: CD90, CD146, and STRO1.

All populations were used in subsequent experiments, which were carried out in triplicate to the passage 4.

-Experimental groups

The enriched populations of the markers were called positive, and non-enriched, negative. From the isolation of cells, for pulp fand scaps following groups were then established: CD90 + -, CD146 + - and STRO1 + -.

For all groups, 1x104 cells/ml were placed in 12-well plates. After 3, 6, 9, and 12 days, the cells were removed from 3 wells at a time by means of TrypLETM reagent action (Gibco, # 12563) and counted in a Neubauer chamber.

-Study of morphological features

In all groups, a cell culture plate containing an opaque background with openings for light was used. This fund contains casting balls (6mm each). Thus, all plates were imaged in the same regions and the openings for the light had the same size. The snapshot characteristics, such as distance from the focus and intensity of light exposure time and gain, were standardized and controlled. All photos were taken at 10X magnification.

Then, the cell area and perimeter measurements were accomplished using the program ImageJ software. Therefore, the outline of the cytoplasm and cell nucleus was done manually using a drawing tool, and then obtaining the area value, and perimeter of such structures. 48 fields were measured, totaling 357 cells. This measurement was performed 24 hours after cell separation by a single operator without knowledge of the analyzed group. The calculation of the cells’ form factor was made from the ratio area perimeter the most circular cells more irregular.

-Synchrotron radiation data collection and procedure

The following protocol was initially validated for the cells of interest in this study (23).The polychromatic radiation emitted by the D09B magnetic dipole of the LNLS-UVX machine was focused by a pair of elliptical-shape mirrors in the so-called Kirkpatrick-Baez conFiguration to an area of 20 microns in diameter . X-ray fluorescence as well as scattered radiation coming from samples were collected at 90 degrees from the incoming beam direction with a Silicon Drift Detector (KETEK GmbH; 140 eV (FWHM) at 5.9 keV). Samples were deposited on thin films and a raster scanning procedure of the sample through the x-ray microbeam was followed to cover its whole area.

All XRF spectra were fitted and processedby the PyMca software (29) for determining the peak area and the elemental concentration of several detected elements. Calibration of the spectrometer was done with a set of pure thin films from Micromatter™ standards (http://www.micromatter.com) and the fundamental parameter method was used for quantitative analysis (30).

-Statistical analysis

Data on the morphology and average fraction of the chemical elements detected by synchrotron radiation have followed a non-parametric curve. The morphology data are presented as the mean, standard deviation, median, minimum, and maximum values. Due to the wide variation of the elemental concentrations values obtained at each measured point of the sample , only the average was adopted in the charts for easy viewing. The groups were compared by the analysis of variance Anova, followed by the Tukey test (for parametric data), and Kruskal-Wallis analysis followed by Dunn’s test (for non-parametric data). For comparisons between populations (enriched or not), adopted Mann-Whitney test was used as the marker. The significance level was 5%.

## Results

**Figure 1:**
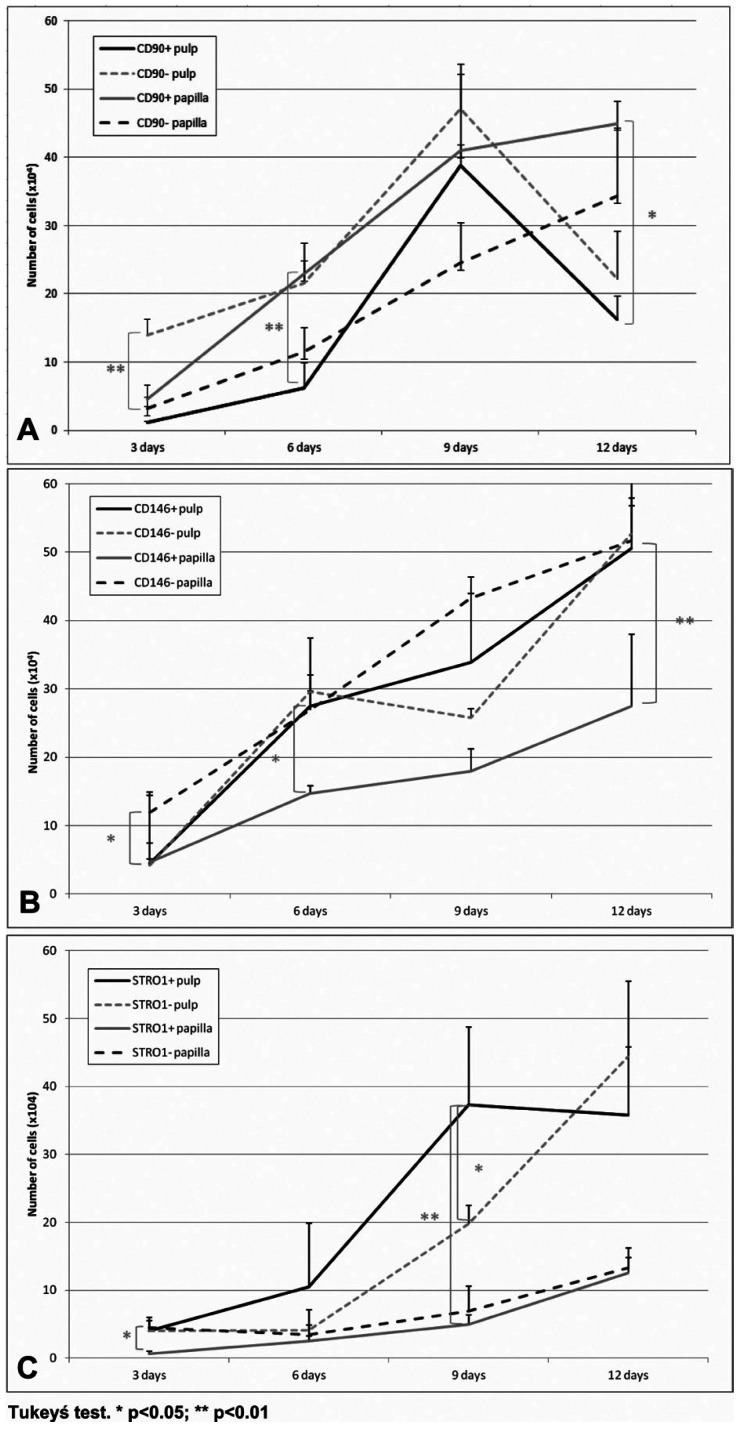
The mean (± standard deviation) of the cell number for each group and the experimental period. A: growth curve for CD90 + and CD90- populations. B: Growth curve for CD146 + and CD146- populations. C: Growth curve for STRO1 + and STRO1- populations.

Figure 1 shows the mean number of positive cells for each marker, derived from the pulp and the papilla, at each time of observation. Aside from the STRO1 pulp, non-enriched populations exhibited faster growth compared to enriched, both in the flesh and in the papilla. The CD90 + population in papilla grew faster than the pulp CD90 + (Tukey’s test, *p* <0.01 for 6 days, and *p* <0.05 for 9 days). The CD146 + derived from the pulp population grew faster than that arising from the papilla, with significant differences at 6 and 12 days (Tukey test, *p* <0.05 for 6 days and *p* <0.01 for 12 days). Regarding STRO1, even though the large variation of the data-positive cells derived from the pulp also grew faster than those that were papilla derived, with significant differences at 9 days (Tukey’s test, *p* <0.05).

Comparing the growth rate between markers in the pulp, the CD146 + population grew faster, with significant differences in the CD90 + 6 days (Tukey test, *p* <0.05). In the papilla, the CD146 + cells exhibited slower growth from CD90 +, with significant differences in 9 days (Tukey test, *p* <0.01). There were no significant differences between the growth of STRO1 + cells compared to positive for the other two markers, both in the flesh and in the papilla.

**Figure 2:**
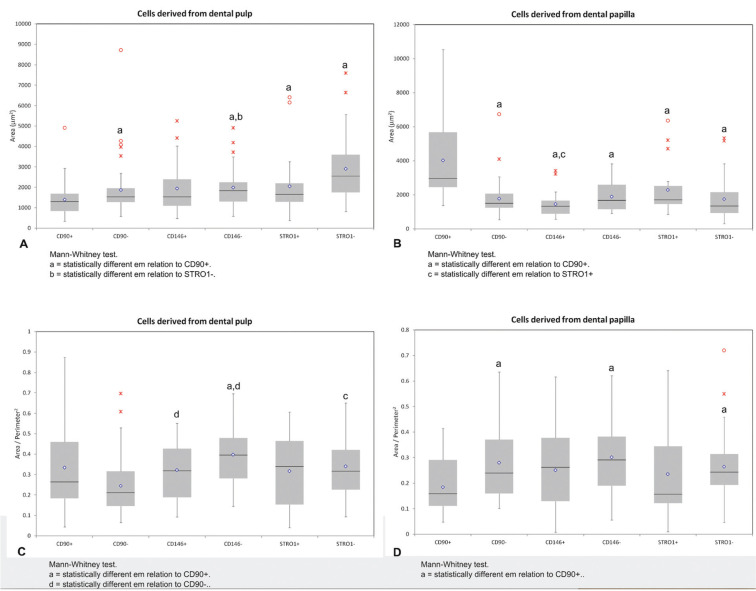
The data on the area and the form factor of the cells from each experimental group.

The analysis of morphometric cellular in Figure 2 shows the data on the area and the form factor of the cells from each experimental group. In the pulp, the lower area was observed in CD90 + cells, with significant differences for CD90- cells (Mann-Whitney test, *p* = 0.02), CD146- (*p* <0.001) + STRO1 (*p* = 0.01) and STRO1- (*p* <0.001). In addition, CD90 + cell pulp exhibited more circular (larger form factor) in relation to CD146- cells (*p* <0.001). In the papilla, the CD90 + cells exhibited a larger area with significant differences from the CD146 +, CD146-, STRO1 + and STRO1- (for all intersections, *p* <0.001). These CD90 + cells of the papilla exhibited even more irregular shape (smaller form factor), being similar to STRO1 + but statistically differing from all of the negative cells (for all intersections, *p* <0.001). Comparison of pulp and papilla noted significant differences only for CD90 +, when the pulp were significantly lower in relation to the papilla (*p* <0.001). For CD146 + and STRO1 + cells, there were no differences in the comparison between the two tissues.

-Micro X-ray Fluorescence Analysis

**Figure 3:**
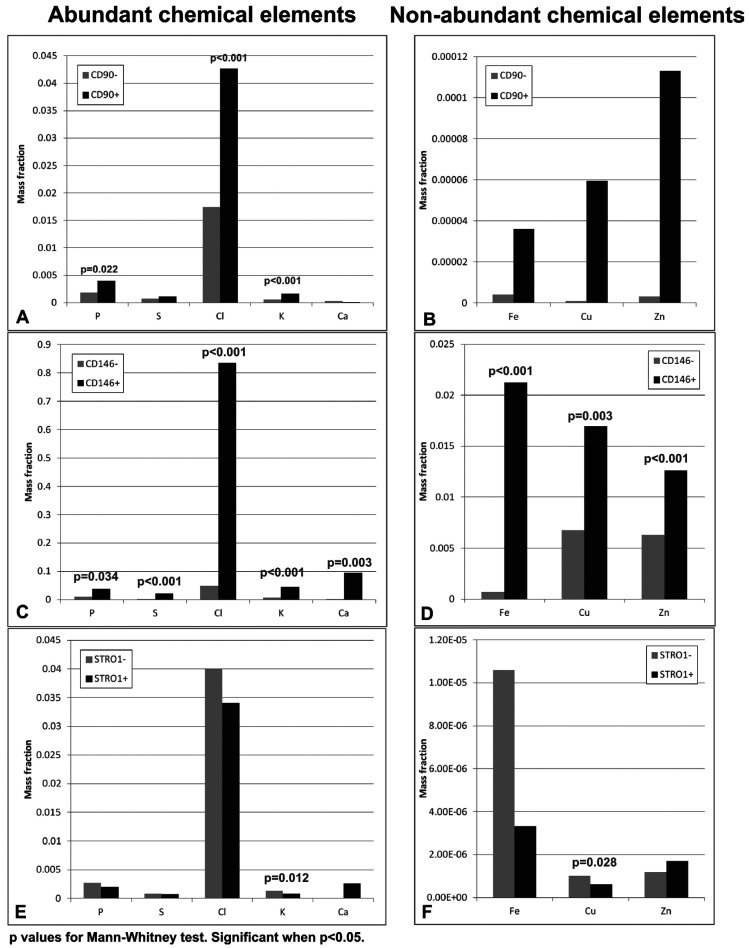
Mean mass fraction for abundant and non-abundant chemical elements present in the cells derived from the dental pulp. A: Cells enriched (+) or not (-) of CD90. B: Cells enriched (+) or not (-) of CD146. C: Cells enriched (+) or not (-) of STRO1-.

Elements such as P, S, Cl, K, and Ca were mosty founded in the analyzed samples whereas Fe, Zn and Cu were less common. Figure 3 contains the average of the ratios of each chemical element in cells derived from pulp. Note that the CD90 + and CD146 + cells exhibited a significantly higher mean mass fraction of P, Cl, and K in relation to the population which was not enriched these markers. The CD146 + cells also showed significantly greater mass fraction of Ca, Fe, Cu, and Zn. The pulp-derived STRO1 + cells did not exhibit a greater amount of any of the elements analyzed in relation to STRO-1.

**Figure 4:**
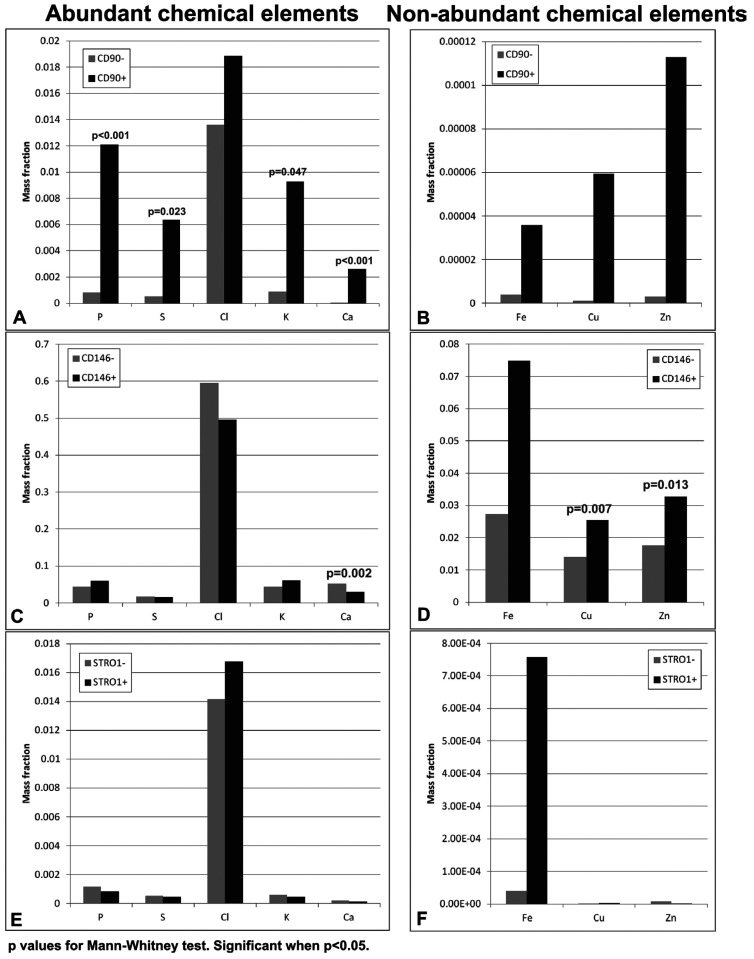
Mean mass fraction for abundant and non-abundant chemical elements present in the cells derived from the scaps. A: Cells enriched (+) or not (-) of CD90. B: Cells enriched (+) or not (-) of CD146. C: Cells enriched (+) or not (-) of STRO1-.

Figure 4 contains the average of the ratios of each chemical element in cells derived from the papilla. The CD90 + cells exhibited a significantly higher amount of P, S, K, and Ca with respect to CD90- cells; with respect to the less abundant elements, despite a greater amount, the differences were not significant. This tendency was similar to that observed for CD90 + cells from the pulp. As for the CD146 + cells derived from the papilla, the amounts were significantly higher only for Cu and Zn, a trend that differed greatly from that observed for the CD146 + cells from the pulp. As for STRO1 + cells, the analyzed chemical elements in general were higher in STRO1- cells, a fact confirmed in the STRO1- cells of the pulp. The only exception was the Fe, which increased in STRO1 + papilla cells in relation to STRO1-, but this difference was not significant.

Comparing the markers, the CD146 + cells exhibited a significantly higher mass fraction of all the elements in relation to the CD90 + cells and STRO1 +, from the pulp and papilla (at all intersections and all elements using the Dunn test, *p* <0.05). An exception was the element S, which did not exhibit significantly different mass fraction compared with CD90 + and CD146 +. A comparison between the CD90 + and STRO1 + cells showed differences only relative to K and S, where the CD90 + originating papilla exhibited a significantly higher mass fraction of these elements than STRO1 + (at all intersections using the Dunn test, *p* <0.05).

## Discussion

In all experiments, we took care to always use the same donor, thus, avoiding possible distortions caused by individual peculiarities. The main result was the finding of a distinct profile among populations enriched for CD90, CD146, and STRO1.

For proliferation, our results showed that pulp derived from the enriched population of CD146 proliferates more than the other. But, analyzing the populations derived from the papilla, the enriched population of CD90 was proliferated most. This result indicates that the CD90 + cells from the pulp probably have a growth potential different from those of papilla CD90 +, and the same may be said for CD146 + cells. Interestingly, the enriched population proliferates STRO-1 markedly less when derived from the papilla, with the same trend when derived from pulp, although in this case the results were not as striking. It is shown that cells enriched by STRO-1 are more proliferative than isolated by CD146 (e.g. (31).), but the donor pool usually gives rise to those populations (10,32,33).

A peculiarity of stem cells is that they have a small size, and this aspect has been associated with greater ease of movement (34). In fact, analysis of cellular morphology in our populations indicated that generally positive cells derived from pulp exhibited a smaller area than the negative. The scaps already exhibit area and a more variable form factor without a clear trend as to the morphology. In the pulp, the smallest area was observed for CD90 + cells. There were no differences compared to CD146 +, while the scaps was for CD146 +. That is to say the assumption is that stem cells have a constant size, regardless of their origin (35,36), was not confirmed in this study. Furthermore, even using the same markers, the differentiation potential of stem cells is varied and generally near the tissue origin of cells, which tend to retain the signature of the tissue in which they originally resided (1,33,37) (38,39); Our results demonstrate that, in general, positive cells exhibited the highest mass fraction of analyzed ions than negative, both the pulp and the papilla. The exception was STRO1, where there were no differences in the enriched populations of this marker. The most common ionic element in the enriched populations were P, S, Cl, and K. This result is in good agreement with another work which used XRF analysis with synchrotron radiation on embryonic pluripotent stem cells during neural differentiation (28). The authors observed P and S polarization during cell differentiation, but no change in the amount of these elements at different stages of differentiation. P and S are considered essential elements for the formation of structural macromolecules (nucleic acid and protein), as well as the energetic metabolism and cellular mechanical functions (40). Its increase has been associated with increased production of RNA, most protein synthesis, and increased proliferation (40,41), whereas the CD90 + cells and CD146 + derived from the pulp exhibited a greater amount of these ions and also higher growth compared to STRO1 +. It is likely that the high metabolism associated with cell proliferation is linked to this higher ionic concentration. Since K and Cl ions are essential elements for the maintenance of intracellular water balance, as well as for the maintenance of pH, intracellular reduction of this ion is an important cellular infeasibility indicator (apoptosis) in stem cells when these cells are cultured *in vitro* (20).

The presence of important metals (Cu, Fe, and Zn) was detected in the aforementioned study (28), in which the metal accumulation was associated with neural differentiation experienced by cells. These metals are considered essential for cellular metabolism, especially for respiration (Cu), proliferation and differentiation (Fe), and synthesis/protein catabolism (42). Particularly Zn, this translocation from the cytoplasm to the nucleus ion was associated with a loss of pluripotency in embryonic stem cells (33). We observed a higher concentration of these important metals in CD146 + cells, but not in other cell populations.

Ca element was significantly increased in the Scap CD90 + population and pulp CD146 +. This element plays a fundamental role in the migration and differentiation of stem cells. There is evidence that stem cells trigger mechanisms that stimulate a Ca2 + influx (43). Furthermore, activation of Ca2 + channels and maintenance of the intracellular Ca2 + gradient is crucial for the activation pathways considered essential for the maintenance of pluripotent stem cells, such as the Wnt pathway and PI3K/Akt (43,44).

The most ionic concentration was generally observed in CD146 + cells. Its growth pattern was different from the others, especially those from pulp, indicating that cells have the capacity of proliferation and particular ionic characteristics. It is worth mentioning that the CD146 + cells exhibited mass fractions of the elements analyzed with different trends considering the pulp and scaps (scaps-only Cu and Zn was different compared to the non-enriched population of this marker), corroborating the particularities of these cell populations with respect to their origin. This was not observed for CD90 + and STRO1 + cells, whose ionic profiles were similar between pulp and papilla.

It is noted that, for any element, the STRO1 + cells exhibited a significantly higher mass fraction with respect to STRO1-. Low ionic concentration of the elements confirms the slower growth of this cell population, indicating a lower metabolic profile. A fact to be considered is that the stem cells are quiescent in tissues only proliferating or exercising any function when stimulated (31,39).

There is evidence that there is wide variation in the elements-traits in stem cells according to the different passages during cultivation, to the extent that these variations are considered important proliferation markers and cell death, especially in relation to the concentrations of P, Cl, K, and intracellular Ca (40,43).

In conclusion, the results of this study showed differences in the proliferation, growth, and ionic profile function in stem cell populations that used the marker and tissue origin of these cells. The cell populations that proliferated more and showed greater ionic concentration were enriched for CD90 and CD146, which were mainly derived from the pulp. Populations that were already derived from STRO1 exhibited lower growth and lower ion concentration, reflecting a lower metabolic pattern. Isolating the stem cells from other cells and analyzing their proliferative potential and differentiation from their ionic profile appears to be promising and clinically applicable. Synchrotron radiation is an effective method for this function, as it enables a complete mapping of intracellular ions without damaging the cells.
